# Volcanic glass properties from 1459 C.E. volcanic event in South Pole ice core dismiss Kuwae caldera as a potential source

**DOI:** 10.1038/s41598-019-50939-x

**Published:** 2019-10-08

**Authors:** Laura H. Hartman, Andrei V. Kurbatov, Dominic A. Winski, Alicia M. Cruz-Uribe, Siwan M. Davies, Nelia W. Dunbar, Nels A. Iverson, Murat Aydin, John M. Fegyveresi, David G. Ferris, T. J. Fudge, Erich C. Osterberg, Geoffrey M. Hargreaves, Martin G. Yates

**Affiliations:** 1 Climate Change Institute, University of Maine, Orono, ME USA; 2 School of Earth and Climate Sciences, University of Maine, Orono, ME USA; 3 College of Science, Swansea University, Swansea, UK; 4 Earth and Environmental Science, New Mexico Institute of Mining and Technology, Socorro, NM USA; 5 Dept. of Earth System Science, University of California, Irvine, CA USA; 6 School of Earth and Sustainability, Northern Arizona University, Flagstaff, AZ USA; 7 Dept. of Earth Science, Dartmouth College, Hanover, NH USA; 8 Earth and Space Sciences, University of Washington, Seattle, WA USA; 9 National Science Foundation Ice Core Facility, Denver Federal Center, Lakewood, CO USA

**Keywords:** Natural hazards, Palaeoclimate

## Abstract

A large volcanic sulfate increase observed in ice core records around 1450 C.E. has been attributed in previous studies to a volcanic eruption from the submarine Kuwae caldera in Vanuatu. Both EPMA–WDS (electron microprobe analysis using a wavelength dispersive spectrometer) and SEM–EDS (scanning electron microscopy analysis using an energy dispersive spectrometer) analyses of five microscopic volcanic ash (cryptotephra) particles extracted from the ice interval associated with a rise in sulfate ca. 1458 C.E. in the South Pole ice core (SPICEcore) indicate that the tephra deposits are chemically distinct from those erupted from the Kuwae caldera. Recognizing that the sulfate peak is not associated with the Kuwae volcano, and likely not a large stratospheric tropical eruption, requires revision of the stratospheric sulfate injection mass that is used for parameterization of paleoclimate models. Future work is needed to confirm that a volcanic eruption from Mt. Reclus is one of the possible sources of the 1458 C.E. sulfate anomaly in Antarctic ice cores.

## Introduction

The second largest sulfate loading event in the last 2500 years has long been thought to originate from the Kuwae caldera in Vanuatu. This event would be second only to the 1259 C.E. Samalas eruption in Indonesia^1^ in sulfate emission, and would have had a similar to or possibly larger impact on the climate system than the 1815 C.E. Tambora eruption^2–4^. Ice core studies first suggested the occurrence of a large volcanic event around 1450 C.E. based on glaciochemical evidence in a South Pole ice core^5^, a large electrical conductivity spike at 1460 C.E. in East Antarctica^6^, and a sulfate increase between 1380–1460 C.E. in a South Pole ice core^7^. The Greenland ice sheet acidity record also shows a modest increase at 1450 C.E.^8^, which was not thought to be a tropical volcanic event at the time. Later, Pang^9^ tied the southern hemisphere ice core anomaly to a large volcanic event from Kuwae volcano in Vanuatu primarily utilizing historical reports and tree ring data. The Kuwae caldera collapse was first suggested to have occurred in 1425 C.E., based on historical reconstructions and radiocarbon dating^10^. The timing was shortly revised by Robin *et al*.^11^ to 1452–1453 C.E. by combining ^14^C and ice core chronologies. Published tree ring frost damage^12^ and density data^13^ were interpreted as being the result of a strong cooling in the northern hemisphere in 1453 C.E., seemingly confirming 1452 C.E. as the start of the major volcanic eruption.

A number of Greenland and Antarctic ice core studies^2,3,14–16^ accepted the 1452 C.E. age and Kuwae as the source caldera. At odds with this are proximal volcanological data collected at Tongoa and Tongariki islands^17^, reported as a sequence of subaqueous deposits with no evidence of widespread fall deposits that would be expected for a large caldera-forming eruption. The same report also disputed early historical evidences, and pointed to early radiocarbon dates^17,18^. This information did not influence the attribution of the largest sulfate signal observed in the developed 2000-yr Law Dome ice core record around 1458 C.E. to the large caldera forming event from Kuwae^19^.

Recently, the *eVolv2k* compilation of stratospheric volcanic injections listed the 1458 C.E. event as the third largest since 500 B.C.E., but Kuwae was not identified as a potential source^20^. Updated tree-ring based paleoclimate reconstructions strongly support a major cooling event in 1453 C.E. in the Northern Hemisphere^21^, implying that a large tropical volcanic event occurred in 1452 C.E. There are no similar reconstructions yet available for the Southern Hemisphere.

**Figure 1:**
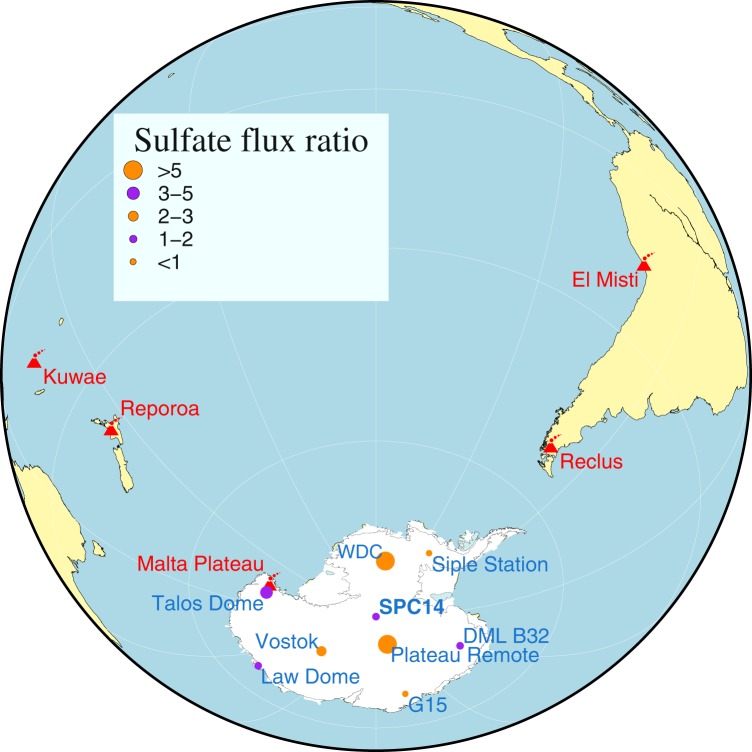
Location map with ice core sites and volcanic centers discussed in this paper. Ice core sites are represented as purple and orange dots. Different colors and sizes are associated with the sulfate flux ratios observed in the ice core records. Possible sources of volcanic products discussed in the paper are marked in red. Mapped using the Generic Mapping Tools software package version 5.4.5.^42^. Grounded ice boundary data from^43^. Sulfate flux values from^3,38,44^ are provided in the Supplementary Table S1.

Recent developments in methodology and instrumentation in the last decade have made it possible to detect and measure physicochemical properties of ultra fine tephra particles (cryptotephra) in various depositional media^22^. The new methodology has made it possible to correlate tephra deposits located thousands of kilometers from source volcanoes^23,24^. Here we test the proposed source of this large 1450s enigmatic sulfate signal by investigating the geochemical composition of cryptotephra (micron sized volcanic glass particles) extracted from ice sampled between 72.53–72.63 meters depth of the South Pole Ice Core (SPICEcore) from Antarctica (89.99°S, 98.16°W; Fig. 1). According to the SP19 timescale^25^, developed using annual layer counting and volcanic signal synchronization with the WDC-06A ice core, this depth interval corresponds to 1458 C.E (Supplementary Fig. S1). To date, no ice-core-based studies have analyzed cryptotephra extracted from the mid-1450s interval. Cryptotephra geochemistry^23^ is instrumental for independent verification of developed time scales and source confirmation of identified volcanic events, which is what this work was originally designed to do.

## Results

**Figure 2:**
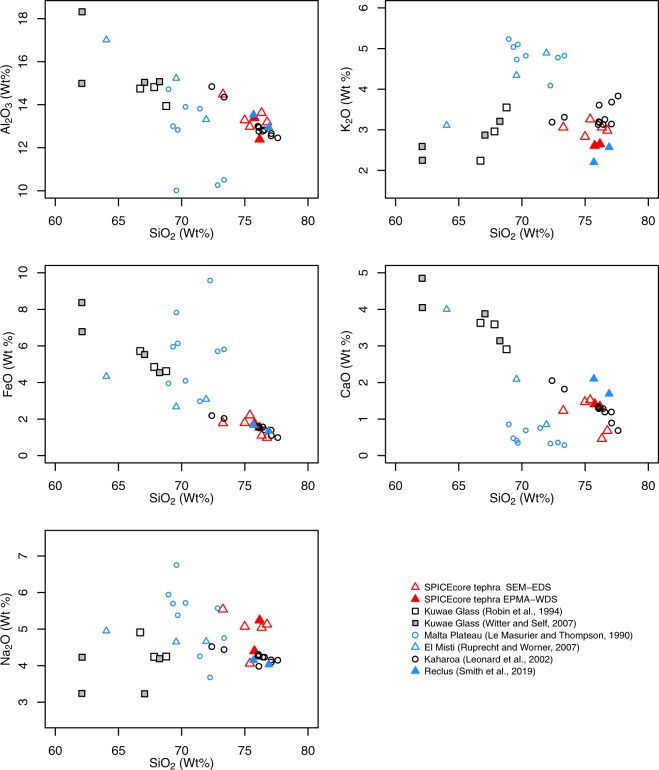
Geochemical composition of individual glass shards extracted from the SPICEcore compared with possible volcanic sources using Harker variation diagrams. Open red triangles represent ice core volcanic glass particle data analyzed using SEM-EDS. The same volcanic glass particles analyzed using EPMA-WDS method are shown with filled red triangles. Symbol size is larger than analytical precision. Tabulated data with SPICEcore cryptotephra and referenced geochemical data are provided in the Supplementary Data section. A subset of this figure is shown in Supplementary Fig. S3.

The particles found in the SPICEcore are rhyolitic with ~75 wt.% SiO_2_, ~3 wt.% Na_2_O and K_2_O, and ~2 wt.% FeO. Figure 2 and Table S2 show the major element concentrations (expressed as oxide weight percent) for two particles measured by EMPA-WDS and for five particles measured by SEM-EDS. All individual point measurements were averaged after normalization of data from both analyses. In previous ice core cryptotephra work studies^26^, a 2% uncertainty for SEM-EDS analyses was established and observed standard deviation of a secondary standard rhyolite glass fit within this parameterization (see Supplemental section for details).

We observed (Fig. 2) slight variations between the two measurement techniques and within populations measured using the same instrument. The SEM-EDS population variability, notably in K_2_O and CaO, can be explained by the particles being unpolished. Rough surface geometry is known to cause some geochemical variations^27^. Iverson and others^26^ established that for some major element oxides, unpolished SEM-EDS measurements are robust enough to be useful for geochemical comparison despite a high standard deviation. In addition, the microprobe results show generally higher Na_2_O wt% compared to the SEM-EDS measurements. This is expected because microprobes are configured to measure Na_2_O in the first counting period during EPMA tephra analyses, whereas all elements are measured simultaneously during SEM-EDS analyses (longer counting time could result in Na loss on the SEM/EDS), and the SEM-EDS measurements are on unpolished particles, which increases variability of the results.

## Discussion

The geochemical composition of volcanic glass particles extracted from 72.53–72.63 meters depth in the South Pole ice core is very distinct from Kuwae eruptive products. The Kuwae glass analyses published by Robin *et al*.^11^ define a basic to intermediate geochemical composition (Fig. 2). The high SiO_2_ observed in ice core tephra particles is inconsistent with any known Kuwae eruptive products. An argument could be made that these particles represent a more evolved composition of the Kuwae glass; however, low K_2_O values do not support this (Fig. 2). The expected trend for the measured ice core cryptotephra SiO_2_/K_2_O ratio is in disagreement with values expected for Kuwae eruptive products, which suggests that the ice core cryptotephra originated from a different volcanic source. The composition of ice core cryptotephra is chemically distinct from Kuwae with a similarity coefficient (SC)^22,28,29^ around 0.5 (see Table S3). In ideal cases, an SC exceeding 0.92 is considered to be a reasonable match^29^. Therefore, other volcanic sources for these particles must be considered.

**Figure 3:**
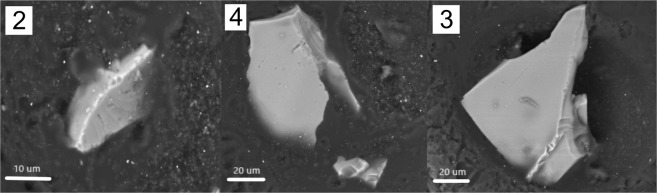
Selected cryptotephra particle images captured from 72.53–72.63 m SPICEcore sample. Each BSE image was taken at 15 kV and 0.5 nA on the Tescan Vega-II XMU before polishing and analysis by EPMA-WDS. Numbers in upper left corners represent the particle number with composition reported in Table S2.

Relatively large particle size (Fig. 3), implies rapid (within weeks after injection into the atmosphere) particle transport, so a range of regional volcanic sources are possible. Typical regional sources for tephra in Antarctic ice cores are Antarctic, sub-Antarctic, South American, and New Zealand volcanoes^30–32^.

The last 1000 years of the New Zealand volcanism record is fairly comprehensive^33^, therefore missing a large eruption from a New Zealand source would be unlikely. One large New Zealand rhyolitic event (Fig. 2) was the Kaharoa eruption which occurred in 1314 C.E. from Taupo Volcanic Zone^34^. Because the tephra was extracted from a relatively modern part of the ice core record, the uncertainty in the layer counting-based SP19 timescale^25^ is relatively low (±3 years). Therefore, the age alone makes Kaharoa tephra an unlikely candidate for the SPICEcore tephra deposits match. In a very comprehensive volcanism record from New Zealand there are no other identified eruptions from New Zealand around the 1450’s, which makes New Zealand volcanism an unlikely source for the tephra extracted from the SPICEcore, despite a very similar geochemical signature (SC range 0.7–0.9, see Table S3) of Kaharoa tephra from the eruption of Mt. Tarawera. The affiliation of tephra with the large sulfate increase also rules out potential transport of wind blown material from New Zealand.

The present characterization of South American and Antarctic volcanism in the 1450’s is less comprehensive than other localities (i.e. New Zealand), and a volcanic eruption from these regions could be a source for SPICEcore tephra deposits. Antarctic volcanism during the last 1000 years typically has a basic composition^35^ and no known rhyolitic eruptions occurred in the 15^*th*^ century in Antarctica. However, the Malta Plateau volcanic area is known for compositionally evolved lavas^35^. A comparison of a general geochemical suite of eruptive products from this area is shown in Fig. 2. It is not a perfect match because FeO values from Malta Plateau volcanic products are within a range of 3–10 wt.%, which is higher than the 1–2.5 wt.% observed in SPICEcore tephra. According to Thouret *et al*.^36^, an eruption was observed in the 1450s C.E. at El Misti volcano in Peru. Geochemical data from this volcanic center are included in Fig. 2 for comparison. The SPICEcore tephra SiO_2_ and K_2_O geochemical fingerprint is inconsistent with the El Misti signature. A geochemical match (SC ranging from 0.7 to 0.8, Table S3) with Reclus tephra and evidence of recent activity of that volcano point to the possibility that a relatively large, unknown volcanic eruption from this volcanic center is responsible for this largest increase in sulfate in Antarctic ice cores in the last two millennia^37^.

The highly variable magnitude and duration of the sulfate peak observed in many Antarctic ice core records (Figs 1, S1) favors a South American source volcanic eruption around 1458 C.E. The 1453 C.E. temperature anomalies in the Northern Hemisphere and the sulfate increase in Greenland ice cores around 1454 and 1460 C.E.^38^ can possibly be attributed to different volcanic eruptions occurring in the northern hemisphere. Several moderate eruptions occurring in both hemispheres over a short time frame could have complex global effects on atmospheric circulation patterns and temperatures^39^. Location sources and potentially different timing for signals attributed to a large volcanic eruption from the Kuwae caldera call for revision of parameterization values for ice core based volcanic forcing time series used in climate models since the pioneering work of^40^.

In addition, the 1458 C.E. Antarctic sulfate signal timing should not be used to adjust the Greenland sulfate signal originally observed at 1452 C.E.^3,12–14,21^. Therefore, the proposed use of a 1458 C.E. event for bipolar synchronization^1^ should be reevaluated.

## Conclusion

This study presents the first quantitative EPMA-WDS and EDS-based geochemical data set on volcanic glass particles extracted from the 72.53–72.63 m depth of the SPICEcore. These new data challenge the majority of previous studies that have suggested that the large sulfate peak present during the ~1450’s C.E. time interval is associated with a large tropical eruption from the Kuwae volcano in Vanuatu, while providing further evidence toward the emerging volcanological and historical data that question such attribution. An accurate determination of the volcanic eruption source and magnitude is important and data presented here suggest that Kuwae has been incorrectly used in past climate model simulations as a source of the ca. 1450s C.E. large sulfate signal in polar ice cores. Although no volcanic source is unambiguously identified in this research, particle size and sulfate appearance in many Antarctic ice core records suggest the eruption is likely affiliated with a tropospherically transported aerosol cloud. A reasonably good geochemical match of SPICEcore tephra with the Reclus volcano and relatively recent, compositionally similar reworked tephra in Laguna Potrok Aike, provide a basis for a preliminary conclusion that possibly an unknown Reclus eruption is responsible for the volcanic signal in Antarctic ice cores. These data contribute to the Antarctic tephra framework needed for future tephrochronological studies, and suggest that paleoclimate models using Kuwae as a climate-forcing event for the ca. 1450 C.E. climate anomaly need to be revised.

## Methods

The sampled interval targeted to locate and extract tephra associated with the 1450s sulfate anomaly was initially selected based on the preliminary version of the SP19 timescale. We sampled two depth intervals: 72.43–72.525 m (sample AntT-335) and 72.525–72.63 m (AntT-336). The sampling was guided by the age of an electrical conductivity (ECM) anomaly in the SPICEcore^41^. In the final version of the SP19 time scale, developed using ECM, visual layer counting, sodium and magnesium ions measurements, interpolation between 251 stratigraphic volcanic tie points to the WD2014 chronology, and previously published South Pole ice core records, the increase in electrical conductivity starts around 1459 (±2) C.E. and lasts for two years^25^. The remarkable manifestation of the sulfate-based volcanic record at the South Pole is attributable to low contributions of sulfate from dust, biogenic, and anthropogenic sources as well as stratospheric air masses^5^. Supplementary Figs S1 and S2 highlight the studied SPICEcore interval and correlated volcanic signals in other Antarctic ice core records.

Using improved ice core tephra mounting methodologies (see online Supplementary section for more details), five tephra particles (ca. 5–50 *μ*m in size) were captured and analyzed using a Tescan Vega-II XMU scanning electron microscope (SEM) equipped with an EDAX Apollo SSD40 energy dispersive spectrometer (EDS). The particles were analyzed on a single spot with a 140 pA beam for 100 seconds of live time at 15 kV of accelerating voltage. Major element oxides were calculated and adjusted using the semi-quantitative EDAX Genesis PhiRhoZ internal quantification procedure and the USNM 72854 VG-568 rhyolitic glass standard. The two largest particles were then polished and subsequently reanalyzed using a Cameca SX-100 electron microprobe equipped with five wavelength dispersive spectrometers (WDS). All measurements were made using instrumentation at the University of Maine. Analytical settings are discussed in Supplementary methods.

## Data Availability

All analytical cryptotephra geochemical data shown in Figs 2 and S3 are available in the Supplement Table S2. The data that support findings of this study are available within the paper and its supplementary files. All tephra related microbeam measurements will be archived with the publication of this paper at the U.S. Antarctic Program Data Center (USAP-DC) http://www.usap-dc.org.

## Supplementary information

Supplementary materials

Table S1

Table S2

Table S3

Dataset Figure 1

Dataset Figure 2 and S3

## References

[1] Sigl M (2015). Timing and climate forcing of volcanic eruptions for the past 2,500 years. Nature.

[2] Cole-Dai J, Mosley-Thompson E, Wight S, Thompson L (2000). A 4100-year record of explosive volcanism from an East Antarctica ice core. Journal of Geophysical Research.

[3] Gao C (2006). The 1452 or 1453 A.D. Kuwae eruption signal derived from multiple ice core records: Greatest volcanic sulfate event of the past 700 years. Journal of Geophysical Research-Atmospheres.

[4] Witter JB, Self S (2006). The Kuwae (Vanuatu) eruption of AD 1452: potential magnitude and volatile release. Bulletin of Volcanology.

[5] Legrand MR, Kirchner S (1990). Origins and variations of nitrate in south polar precipitation. Journal of Geophysical Research.

[6] Moore JC, Narita H, Maeno N (1991). A continuous 770-year record of volcanic activity from east Antarctica. Journal of Geophysical Research-Atmospheres.

[7] Delmas, R. J., Kirchner, S., Palais, J. M. & Petit, J. R. 1000 years of explosive volcanism recorded at the South Pole. *CNRS, Lab. de Glaciologie et Geophysique de l’Environnement, St.-Martin-d’Heres, France* (1992).

[8] Hammer CU, Clausen HB, Dansgaard W (1980). Greenland ice sheet evidence of post-glacial volcanism and its climatic impact. Nature.

[9] Pang KD (1993). Climatic impact of the mid-fifteenth century kuwae caldera formation, as reconstructed from historical and proxy data. Eos Trans. AGU.

[10] Monzier M, Robin C, Eissen J-P (1994). Kuwae (≈1425 A.D.): the forgotten caldera. Journal of Volcanology and Geothermal Research.

[11] Robin C, Monzier M, Eissen J-P (1994). Formation of the mid-fifteenth century Kuwae caldera (Vanuatu) by an initial hydroclastic and subsequent ignimbritic eruption. Bulletin of Volcanology.

[12] LaMarche VC, Hirschboeck KK (1984). Tree rings and volcanoes. Nature.

[13] Briffa KR, Jones PD, Schweingruber FH, Osborn TJ (1998). Influence of volcanic eruptions on northern hemisphere summer temperature over the past 600 years. Nature.

[14] Zielinski GA (1994). Record of Volcanism Since 7000 B.C. from the GISP2 Greenland Ice Core and Implications for the Volcano-Climate System. Science.

[15] Langway CC, Osada K, Clausen HB, Hammer CU, Shoji H (1995). A 10-century comparison of prominent bipolar volcanic events in ice cores. Journal of Geophysical Research.

[16] Ferris DG, Cole-Dai J, Reyes AR, Budner DM (2011). South Pole ice core record of explosive volcanic eruptions in the first and second millennia A.D. and evidence of a large eruption in the tropics around 535 A.D. Journal of Geophysical Research.

[17] Németh K, Cronin SJ, White JDL (2007). Kuwae Caldera and Climate Confusion. The Open Geology Journal.

[18] Oppenheimer C (2003). Ice core and palaeoclimatic evidence for the timing and nature of the great mid-13th century volcanic eruption. International Journal of Climatology.

[19] Plummer C (2012). An independently dated 2000-yr volcanic record from Law Dome, East Antarctica, including a new perspective on the dating of the c. 1450s eruption of Kuwae, Vanuatu. Climate of the Past Discussions.

[20] Toohey, M. & Sigl, M. Volcanic stratospheric sulphur injections and aerosol optical depth from 500 BCE to 1900 CE. *Earth System Science Data Discussions* 1–40 (2017).

[21] Esper J, Buntgen U, Hartl-Meier C, Oppenheimer C, Schneider L (2017). Northern Hemisphere temperature anomalies during the 1450s period of ambiguous volcanic forcing. Bulletin of Volcanology.

[22] Lowe, D. J. *et al*. Correlating tephras and cryptotephras using glass compositional analyses and numerical and statistical methods: Review and evaluation. *Quaternary Science Reviews* (2017).

[23] Pyne-O’Donnell, S. D. F. *et al*. Quaternary Science Reviews. *Quaternary Science Reviews* (2016).

[24] Haflidason, H., Regnéll, C., Pyne-O’Donnell, S. D. F. & Svendsen, J. I. Extending the known distribution of the Vedde Ash into Siberia: occurrence in lake sediments from the Timan Ridge and the Ural Mountains, northern Russia. *Boreas* (2018).

[25] Winski, D. A. *et al*. The SP19 Chronology for the South Pole Ice Core – Part 1: Volcanic matching and annual-layer counting. *Climate of the Past Discussions* (2019).

[26] Iverson NA, Kalteyer D, Dunbar NW, Kurbatov AV, Yates M (2017). Advancements and best practices for analysis and correlation of tephra and cryptotephra in ice. Quaternary Geochronology.

[27] Goldstein, J. I. *et al*. *Scanning Electron Microscopy and X-Ray Microanalysis*, 3rd edition edn (Springer, New York, NY, 2003).

[28] Borchardt G, Aruscavage P, Millard H (1972). Correlation of the Bishop ash, a Pleistocene marker bed, using instrumental neutron activation analysis. Journal of Sedimentary Petrology.

[29] Froggatt PC (1992). Standardization of the chemical analysis of tephra deposits. Report of the ICCT Working Group. Quaternary International.

[30] Basile I, Petit JR, Touron S, Grousset FE, Barkov N (2001). Volcanic layers in Antarctic (Vostok) ice cores: Source identification and atmospheric implications. Journal of Geophysical Research.

[31] Dunbar NW, Kurbatov AV (2011). Tephrochronology of the Siple Dome ice core, West Antarctica: correlations and sources. Quaternary Science Reviews.

[32] Dunbar NW (2017). New Zealand supereruption provides time marker for the Last Glacial Maximum in Antarctica. Scientific Reports.

[33] Lowe DJ, Blaauw M, Hogg AG, Newnham RM (2013). Ages of 24 widespread tephras erupted since 30,000 years ago in New Zealand, with re-evaluation of the timing and palaeoclimatic implications of the Lateglacial cool episode recorded at Kaipo bog. Quaternary Science Reviews.

[34] Hogg A (2003). A wiggle-match date for polynesian settlement of new zealand. Antiquity.

[35] LeMasurier, W. E. *et al*. (eds) *Volcanoes of the Antarctic Plate and Southern Oceans*, vol. 48 of *Antarct. Res. Ser*. (AGU, Washington, DC, 1990).

[36] Thouret J-C (2001). Geology of El Misti volcano near the city of Arequipa, Peru. Bulletin of the Geological Society of America.

[37] Smith RE (2019). Refining the Late Quaternary tephrochronology for southern South America using the Laguna Potrok Aike sedimentary record. Quaternary Science Reviews.

[38] Cole-Dai J (2013). Two likely stratospheric volcanic eruptions in the 1450s C.E. found in a bipolar, subannually dated 800 year ice core record. Journal of Geophysical Research-Atmospheres.

[39] Schmidt AMZ (2018). Volcanic Radiative Forcing From 1979 to 2015. Journal of Geophysical Research-Atmospheres.

[40] Robock A, Free M (1995). Ice cores as an index of global volcanism from 1850 to the present. Journal of Geophysical Research.

[41] Lilien DA (2018). Holocene Ice-Flow Speedup in the Vicinity of the South Pole. Geophysical Research Letters.

[42] Wessel P, Smith WHF, Scharroo R, Luis J, Wobbe F (2013). Generic Mapping Tools: Improved Version Released. Eos Trans. AGU.

[43] Bindschadler R (2011). Getting around antarctica: new high-resolution mappings of the grounded and freely-floating boundaries of the antarctic ice sheet created for the international polar year. The Cryosphere.

[44] Osipov EY (2014). High-resolution 900 year volcanic and climatic record from the Vostok area, East Antarctica. The Cryosphere.

