# Supplementary material for: Volcanic glass properties from 1459 C.E. volcanic event in South Pole ice core dismiss Kuwae caldera as a potential source
Source: Sci Rep. 2019 Oct 8;9:14437. doi: 10.1038/s41598-019-50939-x (PMC6783439; doi:10.1038/s41598-019-50939-x)
Supplement: Supplementary file 1 — Supplementary materials [file 41598_2019_50939_MOESM1_ESM.pdf]

# Volcanic glass properties from 1459 C.E. volcanic event in South Pole ice core dismiss Kuwae caldera as a potential source

Supplementary part

Laura H. Hartman[1], Andrei V. Kurbatov *[1,2], Dominic A. Winski[1], Alicia M. Cruz-Uribe[2], Siwan M. Davies[3], Nelia W. Dunbar[4], Nels A. Iverson[4], Murat Aydin[5], John M. Fegyveresi[6], David G. Ferris[7], T.J. Fudge[8], Erich C. Osterberg[7], Geoffrey M. Hargreaves[9], and Martin G. Yates[2]

[1] *Climate Change Institute, University of Maine, Orono, ME*
[2] *School of Earth and Climate Sciences, University of Maine, Orono, ME*
[3] *College of Science, Swansea University, Swansea, UK*
[4] *Earth and Environmental Science, New Mexico Institute of Mining and Technology, Socorro, NM*
[5] *Dept. of Earth System Science, University of California, Irvine, CA*
[6] *School of Earth and Sustainability, Northern Arizona University, Flagstaff, AZ*
[7] *Dept. of Earth Science, Dartmouth College, Hanover, NH*
[8] *Earth and Space Sciences, University of Washington, Seattle, WA*
[9] *National Science Foundation Ice Core Facility, Denver Federal Center, Lakewood, CO*

*Corresponding Author: akurbatov@maine.edu

# Supplementary information

## Mounting Methodology

This study utilizes a refined methodology for extraction of cryptotephra particles from ice core samples that is based on [1]. The ice core is decontaminated and sectioned in an ultra-clean freezer laboratory into samples following a yearly sampling regime determined by the timescale. The individual samples are then melted in a clean 0.7 L Whirl-Pak bag. Meltwater is transferred into centrifuge vials and spun at 7000 rpm for 15 minutes. One milliliter of sample from the bottom of each centrifuge tube is then evaporated on a pre-heated hot plate at 60-70°C inside of a specially manufactured sample ring mount holder. The water is slowly deposited inside a mount to the glue side of a piece of single-sided Kapton tape, which is supported by an ultra-flat metal surface. Once all water is evaporated for the selected sample, the ring mount is back-filled with Buhler Epo–Thin 2 epoxy resin and hardener, and left overnight to cure under room temperature. Once the epoxy is cured, the mount is detached from the tape, with all embedded particles exposed at the surface. Previous test experiments have shown that no chemical residue remains from the interaction between the tape adhesive and any insoluble particles. All mounts are carbon-coated prior to analysis.

## Cryptotephra geochemical analysis

**Scanning electron microscopy analysis using an energy dispersive spectrometer (SEM-EDS)** Because most particles were smaller than 10 $\mu$m, sample mounts were not polished before SEM/EDS geochemical analysis. Concentrations of major and minor oxides: $SiO_2$, $TiO_2$, $Al_2O_3$, FeO, MgO, CaO, $Na_2O$, and $K_2O$ were measured via secondary electron beam x-ray microanalysis using the University of Maine's Tescan Vega XMU scanning electron microscope. The sample mounts were coated with a 15nm layer of carbon using an Emitech high vacuum evaporator. Particles were analyzed with a 15kV beam scanned over a 1–2 $\mu$m area using a 40 mm$^2$ EDAX Apollo$^{TM}$ energy dispersive x-ray detector and EDAX Genesis$^{TM}$ software. Each analysis accumulated the x-ray spectra for 100 seconds of live time over one analytical spot, and the net peak intensities were converted to oxide weight percent using a standardless, PhiRhoZ-based correction [2]. An additional EDAX Genesis$^{TM}$ PhiRhoZ correction was calculated for tephra using the Smithsonian Rhyolite Glass (NMNH 72854 VG-568).

**Electron microprobe analysis using a wavelength dispersive spectrometer (EPMA-WDS)** The mount was polished prior to microprobe analysis following [1]. WDS analyses were performed using the University of Maine's SX-100 electron microprobe using a method modified from [3]. In this method, three different analytical conditions were used in order to prevent Na migration, optimize precision, and maximize detection limits of minor elements. Na$K\alpha$

and AlK$\alpha$ were analyzed on a TAP crystal at 15kV, 500pA, and a 6 $\mu$m beam, SiK$\alpha$(TAP), CaK$\alpha$(2 PET), KK$\alpha$(2 PET), MgK$\alpha$(TAP) and FeK$\alpha$(LiF) were analyzed at 15kV, 2nA, and a 6 $\mu$m beam, and FK$\alpha$(2 TAP), PK$\alpha$(2 PET), SK$\alpha$(2 PET), ClK$\alpha$(2 PET), TiK$\alpha$(2 PET), and FeK$\alpha$(LiF) were analyzed at 15kV, 80nA, and a 6 $\mu$m beam. Simple silicate and oxide standards and the matrix correction of [4] were used. Counting times varied from 20 to 80 sec. Smithsonian Rhyolite Glass (NMNH 72854 VG-568) and Smithsonian Basaltic Glass (NMNH 113498-I (Kilauea lava glass A99))were analyzed as reference materials to monitor analytical accuracy and precision.

## Normalized glass analyses on particles extracted from the SPICEcore

EPMA-WDS analyses represent a duplicate analysis of particles analyzed on SEM-EDS and available in the comma separated format (File name: TableS2.csv). Particle size is the maximum diameter measured on specified instrumentation. Particle chemistry from multiple analyses represents the average of the multiple analyses. Standards analyzed are the Smithsonian Rhyolite Glass (NMNH 72854 VG-568) and Smithsonian Basaltic Glass (NMNH 113498-I also called Kilauea lava glass A99). The certified composition for the rhyolite glass is as follows in weight percent: $Na_2O$ (3.75), MgO (0.10), $Al_2O_3$ (12.06), $SiO_2$ (76.71), $K_2O$ (4.89), CaO (0.50), $TiO_2$ (0.12), MnO (0.03), and FeO (1.24). The certified composition for the basaltic glass NMNH 113498-I (A99) is as follows in weight percent: $Na_2O$ (2.66), MgO (5.08), $Al_2O_3$ (12.49), $SiO_2$ (50.94), $K_2O$ (0.82), CaO (9.30), $TiO_2$ (4.06), MnO (0.15), FeOt (13.30), $P_2O_5$ (0.38, and $H_2O$ (0.02). Total: 99.39.

## Dataset Figure1

File: Fig1.gmt, provides “bash” script used to generate Fig. 1. An additional free data set is required to map grounding and hydrostatic lines for the Antarctic Ice Sheet. A link to the data set is provided within the file Fig1.gmt. The file also contains a list of ice cores and coordinates of volcanic centers from the Smithsonian global volcanism program database.

Table: S1 provides a list of data sources for mapping sulfate deposition in Antarctica captured in Fig. 1 from [5–7]

## Dataset Figure2-S3

Folder contains the following files:

**FigHarker.R** is a R programming language script that generates Fig. 2 and S3 plots.

**SPICEcoreTephraSEM.csv** SEM data from the Table AntT-336.csv

**SPICEcoreTephraProbe.csv** WDS data from the Table AntT-336.csv

**KuwaeRobin.csv** Kuwae volcanic glass composition data from [8].

**KuwaeWitterSelf.csv** Kuwae volcanic glass composition data from [9].

**MaltaPlateau.csv** Geochemistry of rocks reported for Malta Plateau from [10].

**ElMisti.csv** Geochemistry data from Table 1 in [11] of the historical [12] El Misti volcano eruption. Samples MIS-02-109 Historic, MIS-02-104-70-112, MIS-02-117>112.

**Kaharoa.csv** Geochemistry of volcanic glass associated with Kaharoa tephra, New Zealand. [13].

**ReclusSmith.csv** Geochemistry of volcanic glass associated with Reclus tephra, South America. [14].

# References


1. Iverson, N. A., Kalteyer, D., Dunbar, N. W., Kurbatov, A. V. & Yates, M. Advancements and best practices for analysis and correlation of tephra and cryptotephra in ice. *Quaternary Geochronology* **40,** 45–55 (May 2017).
2. Brown, J. D. & Packwood, R. H. Methods for quantitative electron probe microanalysis of small particles and thin films. *Applied Surface Science* **26,** 294–305 (1986).
3. Hayward, C. L. High spatial resolution electron probe microanalysis of tephras and melt inclusions without beam-induced chemical modification. *The Holocene* **22,** 119–125 (Jan. 2012).
4. Merlet, C. An accurate computer correction program for quantitative electron probe microanalysis. *Microchimica Acta* **114,** 363–376. ISSN: 1436-5073. `https://doi.org/10.1007/BF01244563` (Dec. 1994).
5. Gao, C. *et al.* The 1452 or 1453 A.D. Kuwae eruption signal derived from multiple ice core records: Greatest volcanic sulfate event of the past 700 years. *Journal of Geophysical Research-Atmospheres* **111,** 12107 (June 2006).
6. Cole-Dai, J. *et al.* Two likely stratospheric volcanic eruptions in the 1450s C.E. found in a bipolar, subannually dated 800 year ice core record. *Journal of Geophysical Research-Atmospheres* **118,** 7459–7466 (Jan. 2013).
7. Osipov, E. Y. *et al.* High-resolution 900 year volcanic and climatic record from the Vostok area, East Antarctica. *The Cryosphere* **8,** 843–851 (May 2014).
8. Robin, C., Monzier, M. & Eissen, J.-P. Formation of the mid-fifteenth century Kuwae caldera (Vanuatu) by an initial hydroclastic and subsequent ignimbritic eruption. *Bulletin of Volcanology* **56,** 170–183 (Aug. 1994).
9. Witter, J. B. & Self, S. The Kuwae (Vanuatu) eruption of AD 1452: potential magnitude and volatile release. *Bulletin of Volcanology* **69,** 301–318 (May 2006).
10. *Volcanoes of the Antarctic Plate and Southern Oceans* (eds LeMasurier, W. E. *et al.*) 487 p. (AGU, Washington, DC, 1990).
11. Ruprecht, P. & Wörner, G. Variable regimes in magma systems documented in plagioclase zoning patterns: El Misti stratovolcano and Andahua monogenetic cones. *Journal of Volcanology and Geothermal Research* **165,** 142 –162. ISSN: 0377-0273. `http://www.sciencedirect.com/science/article/pii/S0377027307001898` (2007).
12. Thouret, J.-C. *et al.* Geology of El Misti volcano near the city of Arequipa, Peru. *Bulletin of the Geological Society of America* **113,** 1593–1610 (Dec. 2001).
13. Leonard, G. S., Cole, J., Nairn, I. & Self, S. Basalt triggering of the c. AD 1305 Kaharoa rhyolite eruption, Tarawera volcanic complex, New Zealand. *Journal of Volcanology and Geothermal Research* **115,** 461–486 (2002).

14. Smith, R. E. *et al.* Refining the Late Quaternary tephrochronology for southern South America using the Laguna Potrok Aike sedimentary record. *Quaternary Science Reviews* **218,** 136–156 (Aug. 2019).

15. McGwire, K. C. *et al.* An integrated system for optical imaging of ice cores. *Cold Regions Science and Technology* **53,** 216–228 (2008).

16. Sigl, M. *et al.* The WAIS Divide deep ice core WD2014 chronology – Part 2: Annual-layer counting (0–31 ka BP). *Climate of the Past* **12,** 769–786. `https://www.clim-past.net/12/769/2016/` (2016).

17. Budner, D. M. & Cole-Dai, J. in *Volcanism and the Earth's Atmosphere* 165–176 (American Geophysical Union (AGU), Washington, D. C., Jan. 2013).

# Figures

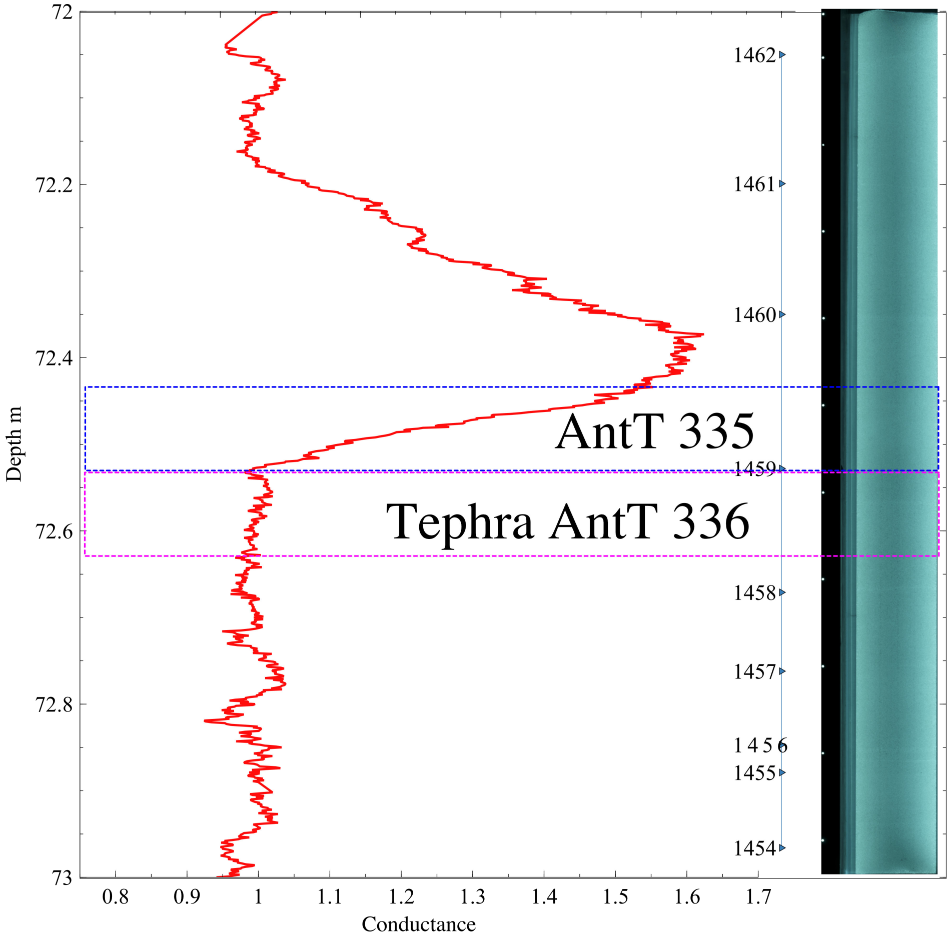


Figure S1: SPICEcore conductance variation with depth. Volcanic glass particles used in this study were found in sample AntT 336. No cryptotephra particles were found in sample AntT 335. An optical scan image [15] acquired during ice core processing at the U.S. NSF Ice Core Facility (on the right) shows no visible tephra deposit at this depth.

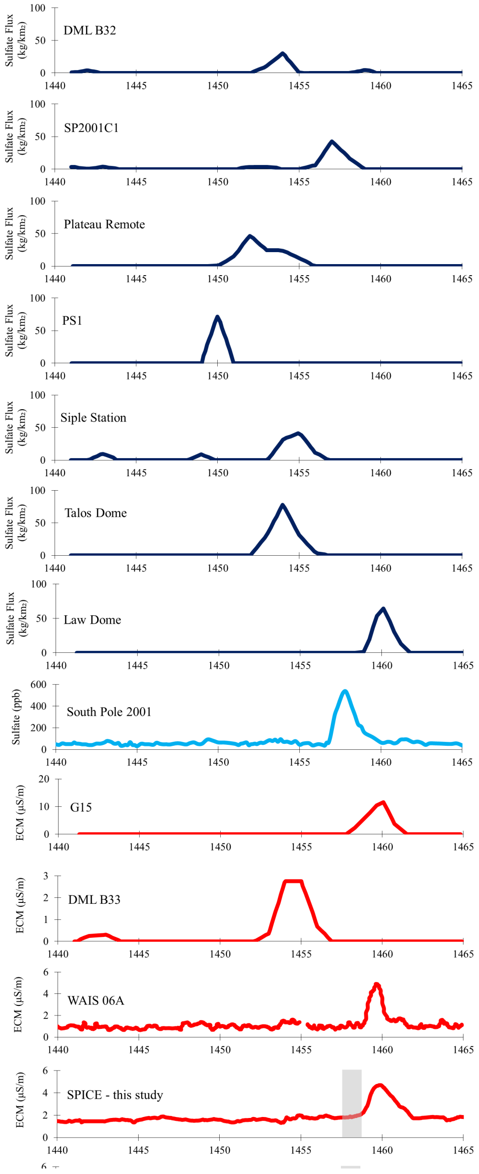


Figure S2: Antarctic glaciochemistry plots for the 1440–1465 C.E. time intervals show a large increase in measured impurities around 1450 C.E. in multiple ice core records. Dark blue plots show sulfate flux in kg/km$^2$, light blue plots show sulfate in ppb, and red plots show ECM in $\mu$S/m for the respective core indicated on each plot. All plots except WAIS 06A [16], South Pole 2001 [17], and SPICE (this study) are based on Figure 3 in [5]. Cryptotephra was extracted from the grey filled section on the SPICEcore subplot.

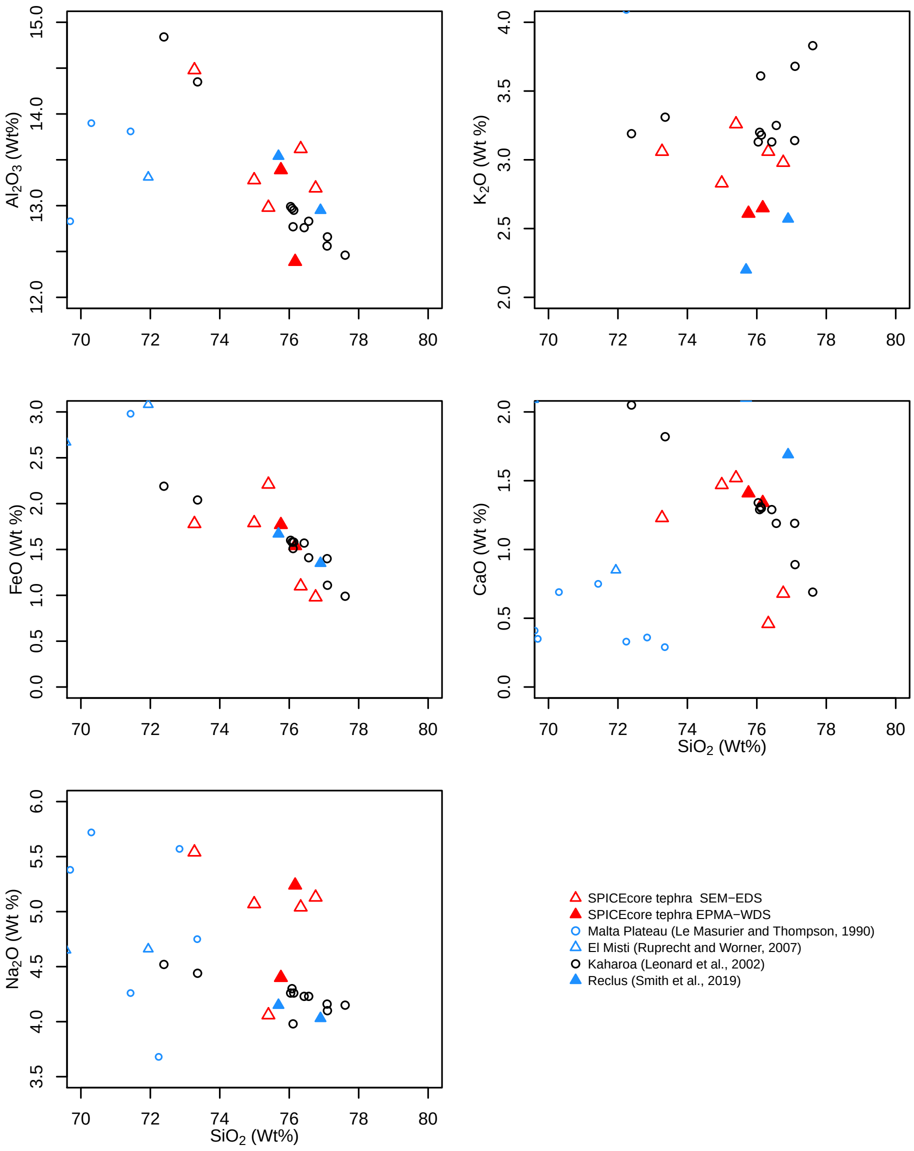


Figure S3: The same as Fig. 2 but zoomed in to $SiO_2$ range 70-80%. The legend and source data are summarized in the original plot.
